# *Pfatp6 *molecular profile of *Plasmodium falciparum *isolates in the western Brazilian Amazon

**DOI:** 10.1186/1475-2875-11-111

**Published:** 2012-04-10

**Authors:** Larissa W Brasil, André LL Areas, Gisely C Melo, Cintia MC Oliveira, Maria G C Alecrim, Marcus V G Lacerda, Connor O'Brien, Walter MR Oelemann, Mariano G Zalis

**Affiliations:** 1 Universidade Federal do Rio de Janeiro, Rua Professor Rodolpho Paulo Rocco, 255, Cidade Universitária, Ilha do Fundão, Rio de Janeiro, RJ, Brazil; 2 Fundação de Medicina Tropical Dr. Heitor Vieira Dourado, Avenida Pedro Teixeira, 25, Dom Pedro, Manaus, AM, Brazil; 3 Universidade do Estado do Amazonas, Avenida Pedro Teixeira, 25, Dom Pedro, Manaus, AM, Brazil; 4 Division of Infectious Diseases, Department of Medicine, Columbia University Medical Center, New York, USA

**Keywords:** Malaria, *Plasmodium falciparum*, Artemisinin, Molecular marker, *pfatp6*, Polymorphisms, Amazon

## Abstract

**Background:**

Anti-malarial drug resistance has emerged as one of the biggest challenges confronting the worldwide effort to control malaria. The appearance of chloroquine and multi-drug resistance had devastating effects on therapeutic efficacy of former first-line agents. Artemisinin has proven to be an excellent therapeutic alternative to fill the void in chemotherapeutic options left by resistance mechanisms. At the time of introduction, no resistance to artemisinins had been recorded, and artemisinins demonstrated excellent parasite reduction rates. In an attempt to protect artemisinin efficacy, the World Health Organization (WHO) made artemisinin-based combination therapy (ACT) its official first-line treatment recommendation for uncomplicated *Plasmodium falciparum *in 2006. In Brazil, artemether/lumefantrine became the Brazilian Malaria Control Programme's official treatment recommendation in 2007. The sarco/endoplasmic reticulum Ca^2+ - ^ATPase ortholog of *P. falciparum *(*pfatp*6) has been suggested as one of the targets of artemisinins. Consequently, *pfatp*6 gene polymorphisms are being investigated as markers of artemisinin resistance elsewhere. The goal of this work was to describe the molecular profile of *pfatp*6 in *P. falciparum *isolates from different localities in the Amazonas State.

**Methods:**

DNA polymorphisms of the *pfatp6 *gene in 80 *P. falciparum *isolates from 11 municipalities of the Amazonas State (Western Brazilian Amazon), before and after the introduction of ACT in the Brazilian anti-malarial guidelines, were analysed by automatic sequencing. Mutations in the *pfatp6 *gene were searched using Mutation Surveyor v3.25 software.

**Results:**

The *P. falciparum pfatp6 *gene presented polymorphisms at codons 37, 630 and 898. The R37K mutation was found in 16% of the samples, A630S in 32% and I898I in 52%. No S769N mutation, however, was detected in the analysed samples.

**Conclusion:**

Despite the small number of samples, data presented here provide baseline information about polymorphisms of *pfatp6 *gene before and after exposure to ACT in a low transmission area, which will help to infer drug selection pressure in this area in the future.

## Background

The Brazilian Amazon Region is responsible for 99.8% of all reported malarial cases in the Brazil, where socioeconomic and environmental conditions favor the proliferation of the *Anopheles darlingi *mosquito. In the State of Amazonas, where this study was conducted, 32,566 cases were reported from January to July in 2010, making it the second most malaria-affected state in Brazil after Pará (51,697 cases). *Plasmodium vivax *accounts for 83.7% of these, but *Plasmodium falciparum *accounts for the majority of the remaining cases [1].

**Figure 1:**
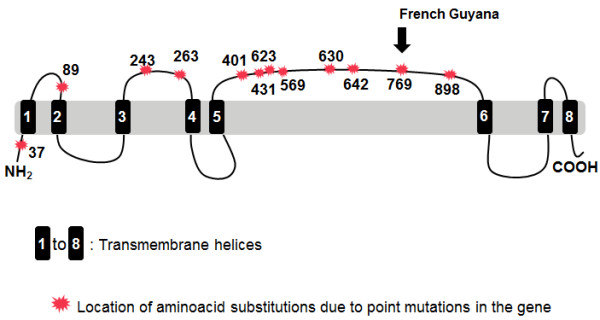
***Pfatpase6*--Membrane protein topology and aminoacid substitutions, evidencing the 769 polymorphism found in samples from the French Guyana (in the Eastern Amazon)**.

*Plasmodium falciparum *resistance to anti-malarial drugs, especially chloroquine and pyrimethamine-sulphadoxine, has emerged as one of the biggest challenges to be faced in malaria control [2]. Artemisinin-based combination therapy (ACT) is now the WHO recommended strategy for preventing the development of drug resistance, and, in Brazil, artemether-lumefantrine was subsequently adopted by the Brazilian Ministry of Health's first-line treatment recommendation for uncomplicated *falciparum *infections. The efficacy of this strategy, however, has become threatened by the discovery of delayed artemisinin sensitivity on the Thai-Cambodian border [3]. The identification and monitoring of indicators of artemisinin resistance is essential for preservation of ACT efficacy [4]. Two genes, originally shown to modulate sensitivity to chloroquine in *P. falciparum*, have been investigated in the context of artemisinin resistance: *pfmdr1 *and *pfcrt*, but failed to correlate with the clinical phenotype of delayed parasite clearance. A previous study, however, with *P. falciparum *suggested that a sarcoplasmic and endoplasmic reticulum Ca^2+^-ATPase (SERCA)-type protein encoded by the gene *pfatp6 *might be the primary target of these drugs [5] (Figure 1), and mutations in this gene may alter *P. falciparum*'s sensitivity to artemisinin.

Jambou et al. reported a significant decrease in *in vitro *sensitivity to artemether in *P. falciparum *isolates from French Guiana, located along Brazil's northern border [6]. This reduced efficacy was associated with a S769N polymorphism in the *PfATPase6 *protein. Although the significance of this mutation has not been determined, these observations could indicate that the S769N mutation could be a marker of drug resistance. French Guyana's proximity to Brazil makes migration of drug resistant parasite populations highly possible [7].

Other polymorphisms have been identified in the *pfatp6 *gene: the double mutation E431K, A623E in Senegal, I89T in Thailand [8], H243Y in Central Africa [9] and T2694 in São Tomé and Principe [10]. In 2008, Dahlstrom et al. [11], studying *P. falciparum *isolates from East and West Africa, identified 33 single nucleotide polymorphisms (SNPs), three of which were found in a frequency higher than 5% in codons H431K, N569K and A630S.

Data on the *pfatp6 *gene in Brazilian isolates is scarce. Recently, three mutations were described in samples from the State of Pará, in nucleotide positions 110, 1916 and 2694 [7]. This study reports *pfatp6 *gene polymorphisms in *P. falciparum *isolates collected before and after the introduction of ACT in eleven endemic municipalities.

## Methods

### Study design

This is an ecological study aimed at evaluating changes in the molecular profile of *P. falciparum *between two distinct time points: before and after the introduction of ACT in Brazil.

### Samples

**Figure 2:**
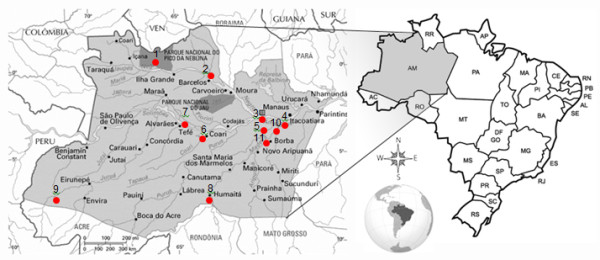
**Map of the Amazonas State showing the municipalities where samples originated (1. São Gabriel da Cachoeira; 2. Barcelos; 3. Manaus; 4. Itacoatiara; 5. Careiro; 6. Coari; 7. Tefé; 8. Humaitá; 9. Guajará; 10. Autazes; 11. Borba)**.

*Plasmodium falciparum *field isolates collected between 2000 and 2005 (60 samples; 10 per year) were obtained from the FMT-HVD Laboratory of Malaria. Samples from between 2009 and 2010 (20 samples; 10 per year) were prospectively collected. Samples from both time points come from the same eleven endemic municipalities of the Amazonas State (Figure 2). Samples were collected as follows: two from Autazes, one from Barcelos, one from Borba, three from Careiro, three from Coari, two from Guajará, two from Humaitá, six from Itacoatiara, 53 samples from Manaus, one from São Gabriel da Cachoeira and six from Tefé. Isolates were collected from patients with uncomplicated *P. falciparum *infections before unsupervised anti-malarial treatment (quinine/doxycycline from 2000 to 2005 and artemether/lumefantrine from 2009 to 2010). As a tertiary care and referral center for infectious diseases in the State of Amazonas, the *Fundação de Medicina Tropical Dr. Heitor Vieira Dourado *(FMT-HVD) routinely follows all patients diagnosed with malaria for 42 days since the middle 1990s. Patients come to the outpatient clinics on days 0, 3, 7, 14, 28 and 42 for clinical evaluation and thick blood smear collection, as part of a protocol to characterize anti-malarial resistance. Diagnosis was carried out by microscopic examination of Giemsa-stained thick blood films as recommended by the Brazilian Malaria Control Programme. After confirming *P. falciparum *mono-infection, 5 mL of venous blood were collected into an EDTA-containing tube. The tube was centrifuged to separate plasma, and erythrocytes were stored at -20°C until DNA extraction.

### DNA extraction

Extraction of DNA from patient blood samples infected with *P. falciparum *was carried out using a Genomic DNA Purification kit (Wizard^®^, USA) according to the manufacturer's protocol.

### PCR amplification and sequencing

Five pairs of oligonucleotide primers (Table 1) were designed for amplification of five genomic sequences between nucleotides 28 to 3078 of the *pfatp6 *gene. The fragments were amplified by PCR and the quality of PCR products generated for each fragment was determined by 1% agarose gel electrophoresis and visualized by staining with ethidium bromide. The DNA concentration was measured by NanoDrop^® ^2000 (Thermo Scientific). The template for DNA sequencing following purification was carried with a commercial kit (SV Gel and PCR Clean-Up System, Wizard^®^, USA).

**Table 1:** Oligonucleotide primers used for PCR amplification and DNA sequencing of *pfatp6 *gene

Primer pair	Sequence 5'→3'	Target region (bps)	Base pairs
1F (sense)	CATACGATGTTGAGGATGTAC	28-722	694
		
2R (antisense)	GACCTATTTCAGTCTTCATACC		

3F (sense)	GAGAATCCTGTTCAGTTGAC	558-1308	750
		
4R (antisense)	ATCCTTCTTCTCCATCATCC		

5F (sense)	ACCGTGTTTCATTTGTTTAGAG	1138-1892	770
		
6R (antisense)	CCATTTGTTGTTGCCTGAGC		

7F (sense)	AATCACCAAGGGGTATCAAC	1771-2467	711
		
8R (antisense)	ACGTATACCAGCCATATGG		

9F (sense)	TTCAAAATATGGGAAAAAGAGCA	2285-3078	805
		
10R (antisense)	TGTTGCTGGTAATCCGTCAG		

Sequencing reactions were carried out using an ABI 3130xl genetic analyzer (Applied Biosystems^®^, USA) as specified by manufacturer's protocol. DNA sequences were generated from both sense and antisense primers, aligned to check for genetic polymorphism and compared to the reference sequence of clone 3D7 (accession PFA0310c in http://www.plasmodb.org). PCR primers and reaction conditions have been published elsewhere [10].

### Data analysis

Sequence quality was assessed using FinchTV^® ^v.1.4.0 (Geospiza^®^) software. Analysis of polymorphisms was performed in Mutation Surveyor^® ^v.3.97 (SoftGenetics^®^, LLC).

### Ethical approval

This study, which used retrospective samples (2000-2005) and prospectively collected samples (2009-2010), received ethical approval from the Ethics Committee Board of the FMT-HVD (protocol 3080-08).

## Results

Samples from 80 patients with acute *P. falciparum *malaria ranging from 13 to 80 years old (71% of which were males) were studied. No instances of treatment failure or recrudescence were observed in the 42-day follow-up period.

**Figure 3:**
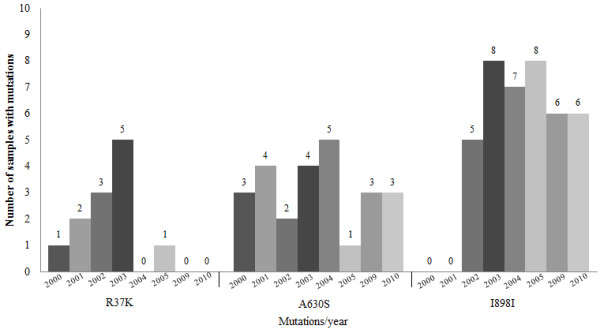
**Polymorphism frequencies by year in the *pfatp6 *gene from samples from different municipalities of the Amazonas State (10 samples analysed per year)**.

PCR amplification and sequencing of *P. falciparum *genomic DNA was successful in all isolates included in this study. A total of three polymorphisms in the *pfatp6 *gene were identified: two non-synonymous (R37K and A630S) and one synonymous (I898I). The mutation at codon 37 lead to the amino acid change Arginine → Lysine. While the second mutation at codon 630 lead to the amino acid change Alanine → Serine. The third mutation at codon 898, however, did not lead to any amino acid change. The prevalence of each mutation was: 16% for R37K, 32% for A630S and 52% for I898I (Figure 3). The G2306A mutation (encoding S769N) was absent in all the samples.

## Discussion

The genetic basis of resistance to anti-malarial drugs can be investigated in several ways. This study describes for the first time a *pfatp6 *gene sequence polymorphism in Amazonas State's samples in two distinct periods: before and after the introduction of artemisinins. No clear change in polymorphism distribution, however, was observed between the two periods. The major limitation of this study was the small number of samples due to the declining number of *P. falciparum *infections. This decline has been particularly pronounced since the introduction of ACTs as first-line treatment in 2007.

A study of the susceptibility to artemisinins and derivatives and molecular analysis of SNPs in Africa, found a silent mutation T2694A in isolates of São Tomé and Príncipe [10]. In this present study, 40 samples from different municipalities presented this mutation, and were similarly found to be phenotypically insignificant.

In 2008, Ferreira et al. also published a similar paper with Brazilian samples [7]. They described the analysis of four SNPs in isolates from Pará in nucleotide positions: 110, 1916, 2306 and 2694. In the present study, no mutations were detected in nucleotides 1916 and 2306. The mutations in nucleotides 110 and 2694, however, were observed with frequencies of 16% and 52% respectively.

Polymorphisms similar to the third observed mutation at nucleotide 1888 in codon 630 in 32% of isolates, were detected in Nigerian samples (A630S), with a frequency of 1.1% [12]. The association between the mutation occurrence and external factors (environment and human host) was tested by bivariate analysis and showed no correlation.

The G2306A mutation (encoding S769N), found in the French Guiana (Eastern Amazon) [6] was absent in the present samples. That is probably explained by the fact that not much immigration occurs from that region to the Amazonas State (Western Brazilian Amazon). Actually the Amazon represents a wide and diverse area with distinct transmission scenarios and still with reduced population mobility, which may parallel the low dissemination of locally originated mutations [13].

*Pfmdr1 *copy number was not assessed in this study. Though *pfmdr1 *copy number is well correlated with decreased therapeutic efficacy of many drugs and found in South America, mefloquine-based ACT is not a first-line agent in Brazil. Therefore, *pfmdr1 *is less likely to be under mefloquine pressure, despite the recent observation that *in vitro *response to lumefantrine could also be associated to *pfmdr1 *mutations [14]. More genes, however, in larger samples from a representative portion of the Amazon should be urgently investigated.

Considering the implication of *pfatp6 *in artemisinin resistance, the molecular variability of this gene should be carefully monitored in areas where *P. falciparum *poses a public health threat. Losing artemisinin efficacy would have a devastating effect on malaria control and treatment efforts, because there is no equivalent oral therapeutic. The gravity of these implications warrants intensive phenotypic and molecular monitoring of field isolates.

## Conclusion

*Pfatp6 *S769N mutation, a potential molecular marker of artemisinin resistance, was not present in the few analysed samples, as well as changes in polymorphism prevalence in *P. falciparum *isolates from Amazonas State since the introduction of ACT in 2007. These data provide a baseline level of genetic variation in a potential candidate gene for artemisinin resistance and will be of value in future resistance monitoring studies. Identification and monitoring of polymorphisms and mutations that confer drug resistance to *P. falciparum *is essential to the success of malaria prevention programmes.

## Competing interests

The authors declare that they have no competing interests.

## Authors' contributions

LWB performed laboratory work and drafted the manuscript. ALLA helped in laboratory work and CMCO was responsible for sequencing reactions. GCM, MGCA, MVGL, WMRO, CO and MGZ participated in its design, coordination and elaborated the final version of manuscript. All authors read and approved the final manuscript.
